# A multipurpose immobilized biocatalyst with pectinase, xylanase and cellulase activities

**DOI:** 10.1186/1752-153X-1-16

**Published:** 2007-06-08

**Authors:** Sohel Dalal, Aparna Sharma, Munishwar Nath Gupta

**Affiliations:** 1 Chemistry Department, Indian Institute of Technology Delhi, Hauz Khas, New Delhi 110016, India

## Abstract

**Background:**

The use of immobilized enzymes for catalyzing various biotransformations is now a widely used approach. In recent years, cross-linked enzyme aggregates (CLEAs) have emerged as a novel and versatile biocatalyst design. The present work deals with the preparation of a CLEA from a commercial preparation, Pectinex™ Ultra SP-L, which contains pectinase, xylanase and cellulase activities. The CLEA obtained could be used for any of the enzyme activities. The CLEA was characterized in terms of kinetic parameters, thermal stability and reusability in the context of all the three enzyme activities.

**Results:**

Complete precipitation of the three enzyme activities was obtained with n-propanol. When resulting precipitates were subjected to cross-linking with 5 mM glutaraldehyde, the three activities initially present (pectinase, xylanase and cellulase) were completely retained after cross-linking. The V_max_/K_m _values were increased from 11, 75 and 16 to 14, 80 and 19 in case of pectinase, xylanase and cellulase activities respectively. The thermal stability was studied at 50°C, 60°C and 70°C for pectinase, xylanase and cellulase respectively. Half-lives were improved from 17, 22 and 32 minutes to 180, 82 and 91 minutes for pectinase, xylanase and cellulase respectively. All three of the enzymes in CLEA could be reused three times without any loss of activity.

**Conclusion:**

A single multipurpose biocatalyst has been designed which can be used for carrying out three different and independent reactions; 1) hydrolysis of pectin, 2) hydrolysis of xylan and 3) hydrolysis of cellulose. The preparation is more stable at higher temperatures as compared to the free enzymes.

## Background

There is an increasing trend towards using enzymes for catalyzing biotransformations [1,2]. In view of their high costs (as compared to chemical catalysts), reusable forms of enzymes, called immobilized enzymes, are often used [3,4]. Immobilization is also sometimes accompanied by greater stability of enzymes during storage or operational stability. Immobilization generally consists of linking an enzyme to a solid matrix or entrapping it in a gel. Insoluble enzyme aggregates produced by extensive chemical cross-linking of enzyme dissolved in a solution have been suggested as an alternative approach for obtaining stable and reusable enzyme preparations [5,6]. In recent years, it has been shown that chemical cross-linking of enzyme precipitates produces more robust biocatalysts. These have been called cross-linked enzyme aggregates (CLEAs) [7-9].

An interesting feature of the CLEAs is that these preparations do not require extensive purification of the enzyme activities. In this respect, CLEAs differ from the CLEC™, another form of enzyme aggregates prepared by chemical cross-linking of enzyme crystals [10]. It has been shown that it is possible to form a CLEA that can catalyze more than one reaction. Thus, a CLEA may catalyze a sequence of reactions. Such CLEAs have been called Combi-CLEAs [8,11]. Recently, we suggested that it should be possible to extend this concept to Combi-CLEAs catalyzing non cascade reactions. Prepared from porcine pancreatic acetone powder, a CLEA was described which had lipase, alpha amylase and phospholipase A_2 _activities [11]. Such multipurpose CLEAs would allow "one biocatalyst for many unrelated biological activities". In the present work, we show that this concept is especially useful in the context of crude/commercial preparations of microbial origin. In as much as enzymes are increasingly isolated from microbes, this work shows that a single biocatalyst preparation from microbial sources could catalyze several biotransformations/bioconversions of industrial relevance.

The system chosen is a well-known commercial preparation called Pectinex™ Ultra SP-L that is used in the food processing industry for hydrolyzing pectin [12]. Our earlier work had shown that this preparation is also rich in xylanase and cellulase activities [13,14]. A multipurpose CLEA with substantial activities of pectinase, xylanase and cellulase was prepared and characterized. The other two activites, xylanase and cellulase, also have well known and extensively documented applications in biotechnology [15,16].

## Results and discussion

**Figure 1:**
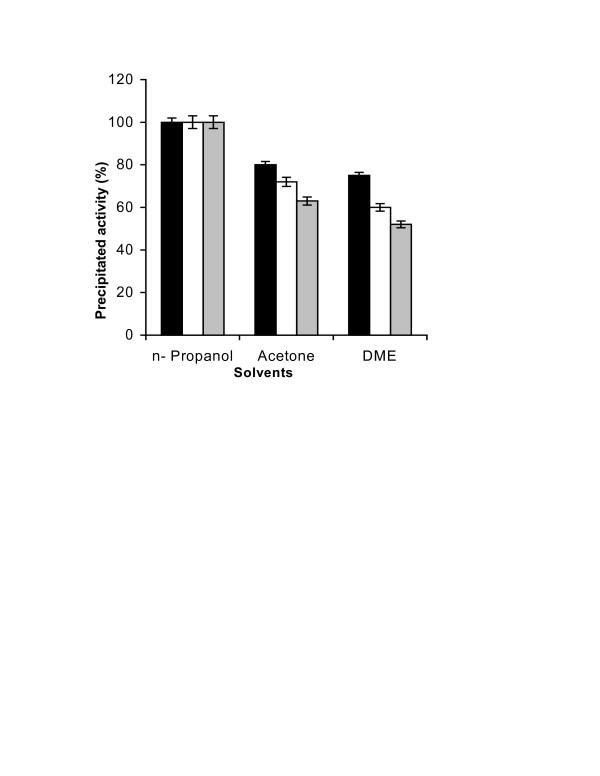
**Precipitation of enzymatic activities with organic solvents: **Three different precipitants (acetone, dimethoxyethane (DME) and n-propanol, 5 ml each) were added to crude Pectinex™ Ultra SP-L (containing pectinase, xylanase and cellulase activity) (1 ml) for complete precipitation of proteins at 4°C as described in the experimental section. The percentage activities of the enzymes in the precipitate were calculated by taking the initial activities as 100%. The experiments were carried out in triplicate and error bars represent the percentage error in each set of readings. -■-: pectinase, -□-: xylanase, --: cellulase.

**Figure 2:**
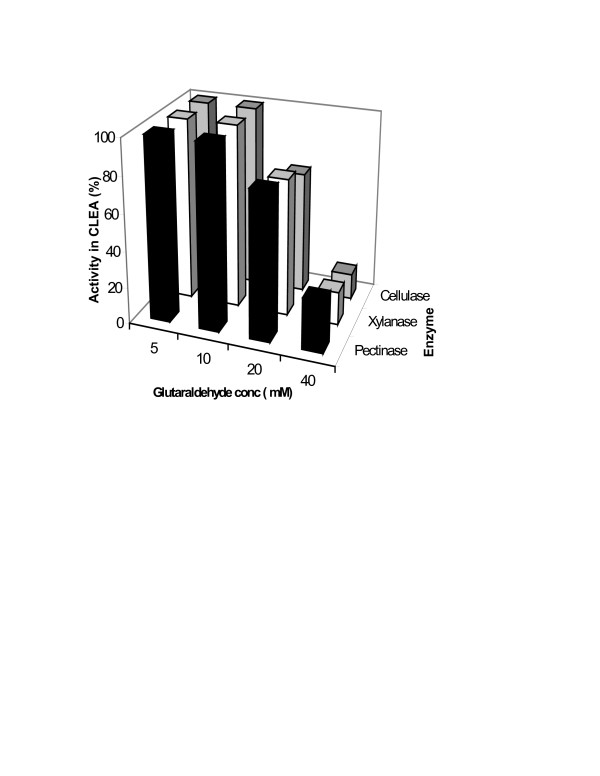
**The Effect on CLEA activity of varying the amount of glutaraldehyde: **CLEA was prepared using varying concentrations of glutaraldehyde (5–40 mM) as described in the experimental section. Experiments were carried out in triplicate and percentage error in each set of readings was within 3%.

The general protocol for the preparation of CLEAs consists of precipitating the enzyme activity by adding salt or an organic solvent [7,9]. This is followed by addition of cross-linking reagent, which is generally glutaraldehyde. Figure 1 shows that n-propanol precipitated all three of the enzymatic activities (viz. pectinase, xylanase and cellulase) completely. Figure 2 shows the effect of varying glutaraldehyde concentration. Following the conditions described in the literature [9,11], 4 h of cross-linking time at 4°C was chosen for this study. As the results show, even a 5 mM glutaraldehyde concentration was good enough to result in CLEA, which retained 100% of all the three enzyme activities originally present in the solution.

The pH optima for the three activities in the CLEA were more or less same as those compared to free enzyme activities. The pH optima were broad (at 4.5 for pectinase and 5.5 for xylanase and 5 for cellulase activities) and agreed with the literature value [17-19]. In the case of cellulase activity, CLEA showed pH optima at 5.5 as compared to 5 observed and reported for free enzyme (Data not shown as figure).

The temperature optima also did not reveal significant changes. The temperature optima observed for free enzymes agreed well with the reported value [17-19]. The pectinase temperature optima was 50°C for both free enzyme and CLEA. Free xylanase showed a temperature optimum of 50°C. CLEA showed broad temperature optima in the range of 50–55°C. Free cellulase has a temperature optimum of 50°C. CLEA showed a broad temperature optima in the range of 50–55°C (data not shown).

Table 1 summarizes the value for Michaelis-Menten parameters obtained with the three activities with free enzyme solution and CLEA. Values of V_max _were found to increase in the case of all three activities measured in CLEA, whereas K_m _values remained more or less unchanged upon CLEA formation. While not much data is available regarding these changes in the kinetic parameters upon CLEA formation, the trends observed in the present case agree with those obtained with CLEA formed from pancreatic acetone powder [11]. It may be mentioned that cross-linking is known to improve marginally the catalytic efficiency of enzyme [20]. Immobilized enzymes generally show increases in K_m _values especially when enzyme activities are assayed with high molecular weight substrates. In the present instance, K_m _values did not increase in a significant manner. Considering that CLEA was formed with just 5 mM glutaraldehyde, perhaps the enzyme aggregates had a very open structure and mass transfer remained as easy as with free enzyme.

**Table 1:** Comparison of kinetic parameters of pectinase, xylanase and cellulase in CLEA

Enzyme	Parameter	Free enzyme	CLEAs
Pectinase	V_max _(mgmin^-1^)	170	207
	K_m _(mgml^-1^)	16	15
	V_max_/K_m _(min^-1^)	11	14
Xylanase	V_max _(mgmin^-1^)	300	400
	K_m _(mgml^-1^)	4	5
	V_max_/K_m _(min^-1^)	75	80
Cellulase	V_max _(mgmin^-1^)	450	500
	K_m _(mgml^-1^)	28	27
	V_max_/K_m _(min^-1^)	16	19

The kinetic parameters were calculated by varying the substrate concentration. The data were fitted in Hanes-Woolf plot using Leonora software [26] as mentioned in the experimental section. The experiments were carried out in triplicate and the percentage error in each set of readings was within 3%.

Table 2 shows the remarkable thermostabilization of the enzymes present in the preparation. In all three of the cases, half-lives have increased upon CLEA formation. Cellulase activity was most thermostable and its thermoinactivation was measured at 70°C. Pectinase was least stable and its thermoiactivation was measured at 50°C. The largest increase in stability was in the case of pectinase in which the half-life increased from 17 to 180 minutes.

**Table 2:** Half-life of pectinase, xylanase and cellulase in CLEA

Enzyme	Temperature (°C)	Half-life (t_1/2_) (minutes)	
		
		Free	CLEAs
Pectinase	50	17	180
Xylanase	60	22	82
Cellulase	70	32	91

The half-life values were calculated by plotting ln(residual activity of enzyme) vs time [19]. In all the three cases, the activity at 0 minute was taken as 100%. The experiments were carried out in triplicate and the percentage error in each set of readings was within 3%.

**Figure 3:**
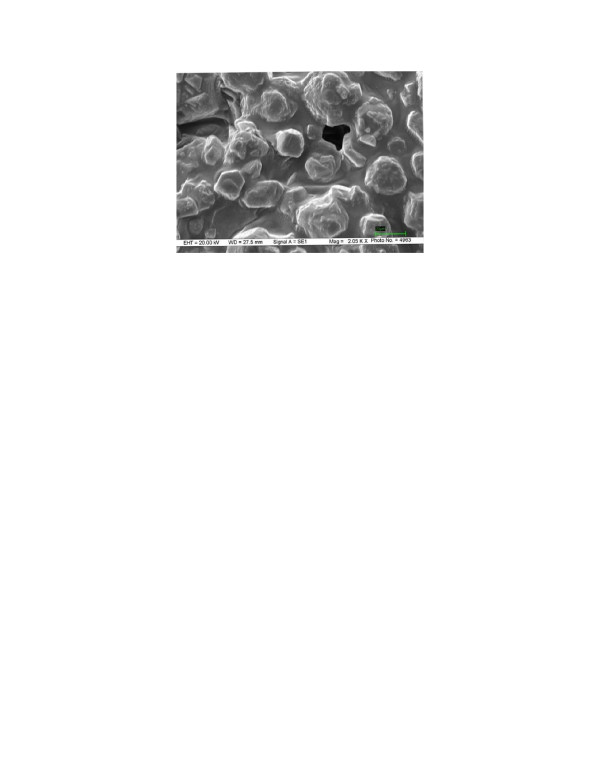
**Scanning electron microscopy (SEM) image of CLEA: **SEM was carried out on Zeiss EVO50 scanning electron microscope, UK. Sample was dried by rinsing with anhydrous acetone, placed on a sample holder, coated with silver before being scanned under vacuum. The particle size was determined from the micrograph with the scale of 20 μm unit.

The Scanning electron micrograph (SEM) of this CLEA preparation is shown in Figure 3. The CLEAs were found to have diameter in the range of 15–30 μm. The size of various CLEAs (and their clusters) has been reported to be in the range of 0.1 μ to 100 μm [7].

**Figure 4:**
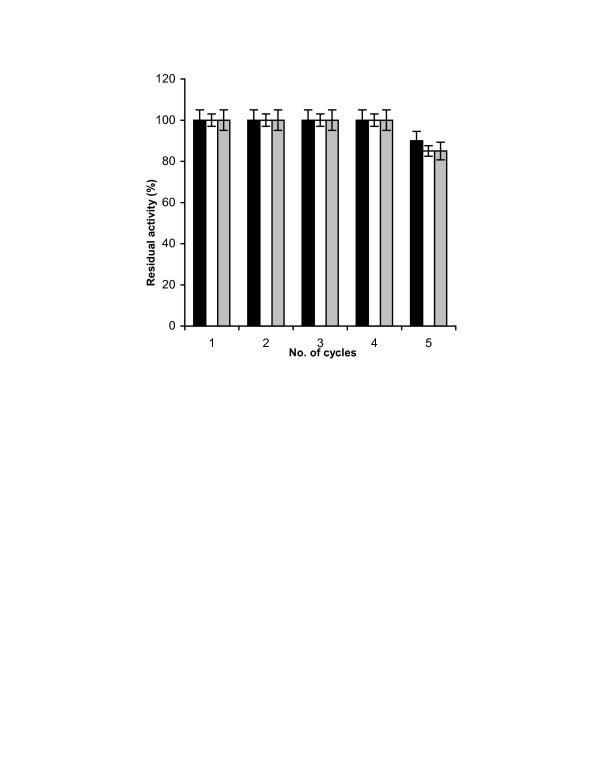
**Reusability of pectinase, xylanase and cellulase in CLEA: **The reusability of pectinase in CLEA was studied by carrying out the hydrolysis of polygalacturonic acid at 30°C for 30 minutes. The reusability of xylanse and cellulase was studied by carrying out hydrolysis of xylan and carboxymethyl cellulose at 50°C for 30 minutes respectively. After each cycle, the reaction mixture was centrifuged at 10, 000 × g and the reducing sugar in the supernatant was measured using 3,5-dinitro salicylic acid. The CLEA recovered after centrifugation was used for next cycle of hydrolysis. The experiments were carried out in triplicate and error bars represent the percentage error in each set of readings.-■-: cellulase, -□-: xylanase, --: pectinase.

Figure 4 shows the reusability of CLEA for all the three enzyme catalyzed reactions. In all the three cases, CLEA could be reused three times without any loss of any of the enzyme activities. After this, slow decline in the activities in all three cases started.

## Conclusion

To conclude, the present work outlines the preparation of a multipurpose robust biocatalyst. The biocatalyst preparation is more thermostable and reusable. The multipurpose biocatalyst can be used for bioconversions (such as hydrolysis of pectin, xylan and cellulose) of considerable biotechnological relevance.

## Experimental

Xylan and polygalacturonic acid were purchased from Sigma Chemical Co., St. Louis, USA. PectinexTM Ultra SP-L (a commercial preparation of pectolytic enzymes from a selected strain of Aspergillus niger) was a kind gift from Dr. J.S.Rao, Novozymes, Bangalore, India. All other chemicals used were of analytical grade.

### Determination of pectinase activity

The activity of pectinase was estimated using polygalacturonic acid as the substrate according to the method described [21]. One unit of enzyme activity is defined as the amount of enzyme required to produce one μmol of galacturonic acid per minute under assay conditions. The amount of galacturonic acid was estimated using the dinitrosalicyclic acid method [22].

### Determination of xylanase activity

The activity of xylanase was estimated using xylan as the substrate [23]. One unit of enzyme activity is defined as the amount of enzyme required to produce one μmol of reducing sugar per minute under assay conditions. The amount of reducing sugar was estimated using the dinitrosalicyclic acid method [22].

### Determination of cellulase activity

The activity of cellulase was estimated using carboxymethyl cellulose as a substrate [24]. One unit of enzyme activity is defined as the amount of enzyme required to produce one μmol of reducing sugar per minute under assay conditions. The amount of reducing sugar was estimated using the dinitrosalicyclic acid method [22].

### Protein estimation

Protein concentration was determined according to the procedure described by Bradford [25] using bovine serum albumin as the standard.

### Precipitation of enzymes by organic solvents

Chilled organic solvents (acetone, dimethoxyethane (DME) and n-propanol, 5 ml each) were added dropwise separately to 1 ml of commercial preparation Pectinex™ Ultra SP-L with shaking and kept for 15 minutes at 4°C for complete precipitation of enzymes and then centrifuged for 5 minutes at 10,000 × g. The supernatant was discarded and the precipitate was redissolved in 0.05 M sodium acetate buffer pH 5. Pectinase, xylanase and cellulase activities were estimated in the solution.

### Preparation of CLEA

Chilled n-propanol (5 ml) was added to the crude enzymatic solution (1 ml) in capped centrifuge tubes. After keeping the mixture for 15 minutes at 4°C for complete precipitation of enzymes, varying amounts of glutaraldehyde were added. The tubes were shaken continuously during the addition. The mixture was kept at 4°C for 4 h with constant shaking at 300 rpm. At the end of the reaction time, the suspension was centrifuged at 10,000 × g for 5 minutes. The supernatant was decanted and the pellets were washed 3 times with 0.05 M sodium acetate buffer at pH 5 to remove unreacted glutaraldehyde. The final enzyme preparation was kept in the same buffer (1 ml) at 4°C.

### Determination of kinetic parameters

The kinetic parameters of free enzymes and CLEA were determined by measuring the initial rates of enzymes with varying amounts of respective substrate solutions under the assay conditions. The data were fitted in Hanes-Woolf equation using Leonora software to calculate the kinetic parameters [26].

### Thermal stability study

Thermal stability was determined for pectinase, xylanase and cellulase present in CLEA. In the case of pectinase, the thermal stability of free enzyme and CLEA was studied at 50°C for 30 minutes and those of cellulase and xylanase were studied at 70°C and 60°C for 60 minutes respectively. In the three cases, the activity at 0 minute was taken as 100%.

### pH and temperature optima of pectinase, xylanase and cellulase

The pH optima of pectinase, xylanase and cellulase in CLEA were studied over the pH range of 3.5–9.5. Temperature optima of pectinase, xylanase and cellulase in free form and in CLEA were studied over the range of 25–70°C.

### Reusability of pectinase, xylanase and cellulase

To evaluate the reusability of pectinase, xylanase and cellulase in CLEA, the CLEA in each case was washed with the assay buffer after each use and then suspended again in a fresh reaction mixture to measure enzyme activity. The residual activity was calculated by taking the enzyme activity of the first cycle as 100%.

## Authors' contributions

MNG suggested the idea about multipurpose CLEA, identified the source of multiple enzyme activities and was actively involved in the discussions of the results. AS optimized the conditions for preparation of CLEA. SD carried out the characterization of CLEA, SEM studies of CLEA and enzyme assays. All authors jointly drafted, proof read and approved final manuscript.
